# Changes in Skeletal Muscle Mass Index With Personalized Exercise and Meal Photo Analysis via Nutrition Applications: Single-Arm Pilot Study

**DOI:** 10.2196/71807

**Published:** 2025-09-25

**Authors:** Hidenori Onishi, Yasutaka Mizukami, Yuki Niida, Kazue Fujita, Osamu Yamamura

**Affiliations:** 1 Department of Community Medicine, Faculty of Medical Sciences, University of Fukui, 23-3 Matsuokashimoaizuki Eiheiji-choYoshida-gun, Fukui, 910-1193, Japan, +81 776618264, +81 776618270; 2 Department of Health and Nutrition, Faculty of Human Life, Jin-ai University, Echizen-City, Fukui, Japan

**Keywords:** Sarcopenia, Skeletal Muscle Index, Exercise, Meal Photos, Dietary Guidance System

## Abstract

This study evaluated changes in the skeletal muscle mass index (SMI) in community residents who received a photos-based dietary guidance system, personalized exercise plans, and meal photography feedback to address sarcopenia. These findings indicated a possible upward trend in SMI within 3 months.

## Introduction

Sarcopenia is characterized by the progressive loss of skeletal muscle mass and function and has become an important health challenge among older adults [1]. Nutrition and exercise have been shown to be effective in preventing and improving sarcopenia [2]. To address regional challenges, remote measurements using Internet of Things (IoT) technology are essential. Our research team is collaborating with private companies to develop an application that provides personalized exercise and nutrition management using tablet devices. This study aimed to evaluate the effectiveness of providing nutrition guidance and exercise programs through IoT technology to improve the skeletal muscle mass index (SMI).

## Methods

### Study settings

Since June 2022, health screenings have been conducted for community-dwelling older adults in Wakasa Town (Fukui, Japan), with 226 participants to date. This retrospective interventional study invited screening participants (June and December 2022, and December 2023) via email [3].

The exclusion criteria included lack of informed consent, hospitalization or residence in care facilities, investigators’ determination that participation was inappropriate, or unsuitability for nutritional management.

The screening procedure included study explanation and obtaining informed consent, medical interview, physical function testing, and body composition analysis.

Assessments included body composition measured using a multifrequency analyzer (MC-780A-N; Tanita). Sarcopenia was classified according to the AWGS 2019 criteria [4] into seven categories. Participants received dietary guidance via an application and personalized exercise plans based on their functional categories. The intervention included tablet training, program provision, and home-based implementation. The application was run on Android 12 tablets.

Prepost comparisons used the Wilcoxon signed-rank test (version 1.54; EZR), with significance set at *P*<.05.

### Ethical Considerations

This study was approved by the Fukui University Medical Research Ethics Review Committee (approval number: 20220046). All participants provided informed consent, and all data used for analysis was anonymized and used under the original consent.

## Results

**Figure 1.**
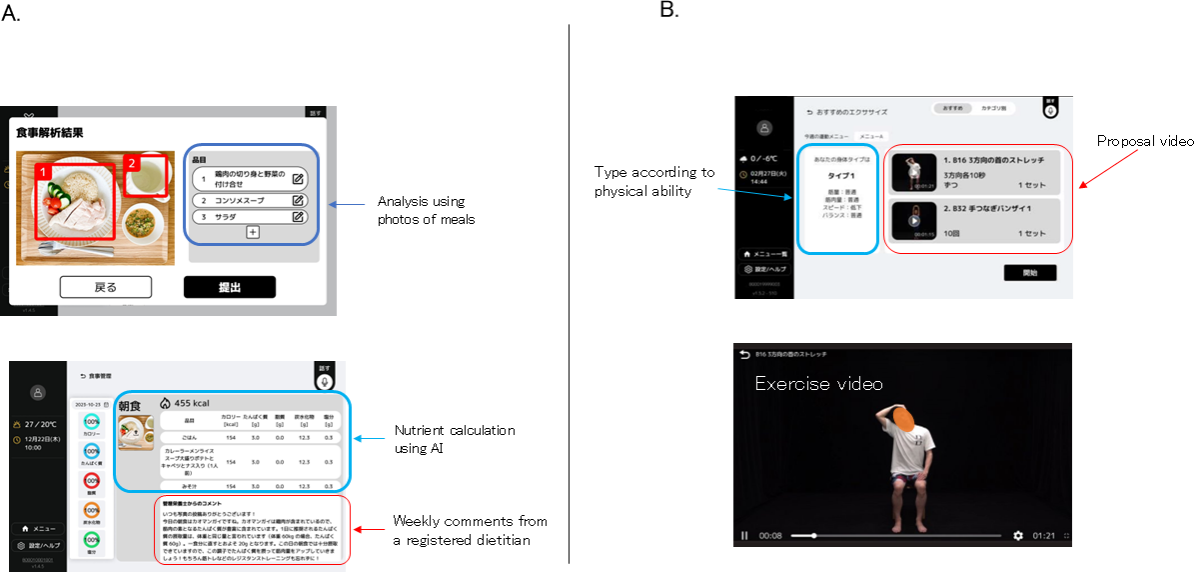
Overview of the developed exercise and nutrition application (A) Automatic analysis of meals using AI and comments from a registered dietitian (B) Proposing personalized videos based on physical ability [3].

This study included 31 participants (14 men and 17 women; mean age: 72.8 y). After excluding 11 participants who met the exclusion criteria, 20 participants were included in this study. The pilot study was conducted from March to June 2024 [3]. Seven exercise programs were implemented at a frequency of 2‐3 times per week. The low-impact resistance training program included video guides [3] (Figure 1A and B). Dietary guidance was provided by having the participants upload photos of their meals and receive remote feedback from registered dietitians. Dietary guidance was defined as “utilized” if the application was used three or more times per week. Additionally, group assignment criteria were based on continuous application use for 7 weeks or more, and participants were categorized into three groups: (1) dropout group with four participants, (2) diet-only group with eight participants, and 3) diet-plus-exercise group with eight participants. No significant differences were observed between the dropout and control groups before or after the intervention. In the diet-only group, significant differences were observed in SMI (*P*=.03) in men and total body muscle mass (*P*=.03) and limb skeletal muscle mass (*P*=.03) in women. In the diet-plus-exercise group, significant differences were observed in total muscle mass (*P*=.009) in women, and in limb skeletal muscle mass (men: *P*=0.01; women: *P*=.009) and SMI (men: *P*=.004; women: *P*=.02) (Table 1).

**Table 1.** Comparison of outcomes before and after using the developed exercise and nutrition application.

Study outcomes	Participants (N=20)									Men (n=9)									Women (n=11)								
	Discontinuation group^a^ (n=4)			Diet-only group^b^ (n=8)			Diet-plus-exercise group^c^ (n=8)			Discontinuation group (n=2)			Diet-only group (n=4)			Diet-plus-exercise group (n=3)			Discontinuation group (n=2)			Diet-only group (n=4)			Diet-plus-exercise group (n=5)		
	Before use	After use	*P* value^d^	Before use	After use	*P* value^d^	Before use	After use	*P* value^d^	Before use	After use	*P* value^d^	Before use	After use	*P* value^d^	Before use	After use	*P* value^d^	Before use	After use	*P* value^d^	Before use	After use	*P* value^d^	Before use	After use	*P* value^d^
Age (years), mean (SD)	77.0 (1.8)	77.5 (2.1)	.35	70.4 (4.2)	70.9 (4.6)	.02	75.4 (8.9)	76.0 (8.8)	.001	77.5 (2.1)	78.5 (2.1)	.35	71.3 (4.3)	72.0 (4.7)	.04	78.0 (8.5)	78.7 (8.5)	.01	76.5 (2.1)	76.5 (2.1)	≥.99	70.3 (4.6)	70.5 (5.0)	≥.99	73.8 (9.7)	74.4 (9.5)	.07
Height, cm	153.0 (8.5)	153.3 (8.3)	.42	161.7 (8.5)	161.4 (8.7)	.04	158.0 (10.1)	158.1 (10.2)	.28	160.7 (2.8)	160.7 (2.8)	≥.99	168.6 (5.9)	168.5 (5.9)	.27	166.9 (10.0)	167.3 (10.1)	≥.99	145.3 (6.7)	145.9 (7.2)	.50	154.9 (5.8)	154.4 (5.8)	.69	152.6 (7.4)	152.6 (7.4)	.72
Body weight, kg	54.8 (9.9)	53.7 (10.9	.13	61.4 (8.7)	61.5 (9.0)	.95	55.2 (12.7)	55.4 (12.8)	.48	63.8 (7.6)	65.3 (9.5)	≥.99	68.9 (4.8)	69.0 (5.0)	≥.99	66.8 (15.9)	66.9 (16.4)	≥.99	45.8 (3.4)	44.0 (3.4)	.50	54.0 (5.5)	54.1 (6.7)	.44	48.1 (5.8)	48.4 (5.8)	.97
BMI, kg/m2	23.2 (1.8)	22.6 (2.4)	.25	23.3 (1.6)	23.5 (1.7)	.78	21.8 (2.7)	21.9 (2.6)	.93	24.7 (2.1)	24.6 (2.8)	≥.99	24.3 (2.0)	24.3 (2.0)	.92	23.7 (3.0)	23.6 (3.3)	.73	21.7 (0.4)	20.7 (0.5)	.50	22.5 (0.8)	22.6 (1.3)	.31	20.7 (2.3)	20.8 (2.2)	.76
Muscle mass - whole body, kg	37.2 (7.5)	37.8 (7.5)	.13	41.9 (7.5)	42.6 (7.8)	.03	38.0 (8.8)	38.2 (8.8)	.11	44.3 (2.9)	44.9 (3.6)	.50	48.9 (2.9)	50.0 (2.6)	.09	46.9 (9.9)	47.2 (10.0)	.08	30.2 (3.9)	30.7 (3.5)	.50	34.9 (3.7)	35.3 (3.6)	.03	32.6 (2.9)	32.9 (2.9)	.009
ASM^e^, kg	15.9 (3.4)	16.4 (3.3)	.13	19.3 (3.8)	19.9 (4.1)	.02	17.1 (4.7)	17.5 (5.0)	.04	18.9 (1.7)	19.4 (2.2)	.50	22.5 (2.1)	23.5 (1.9)	.06	21.3 (6.4)	21.9 (6.6)	.01	12.9 (2.7)	13.5 (2.1)	.50	16.0 (2.6)	16.3 (2.7)	.03	14.7 (1.9)	14.8 (1.9)	.009
SMI^f^ , kg/m2	6.7 (0.7)	6.9 (0.7)	.18	7.3 (0.8)	7.5 (0.8)	.01	6.7 (1.0)	6.9 (1.0)	.02	7.3 (0.4)	7.5 (0.6)	.50	7.9 (0.3)	8.3 (0.3)	.03	7.5 (1.5)	7.7 (1.5)	.004	6.1 (0.7)	6.3 (0.4)	≥.99	6.6 (0.6)	6.8 (0.6)	.06	6.3 (0.3)	6.3 (0.3)	.009
Five-times-sit-to-stand test, s	7.3 (1.8)	8.1 (3.5)	.88	7.5 (1.3)	6.6 (1.5)	.11	6.7 (0.8)	7.0 (1.3)	.95	7.3 (0.6)	7.6 (0.4)	≥.99	7.9 (1.7)	6.5 (1.8)	.16	7.2 (1.0)	7.7 (1.1)	.43	7.4 (3.5)	8.7 (6.8)	≥.99	7.0 (0.9)	6.6 (1.6)	≥.99	6.5 (0.7)	6.6 (1.5)	.83
Standing on one leg with eyes open, s	31.4 (28.6)	28.8 (18.3)	.63	42.9 (18.8)	37.5 (20.5)	.30	39.4 (22.2)	42.2 (23.2)	.58	32.0 (39.6)	27.3 (27.6)	≥.99	38.9 (24.5)	26.2 (23.2)	.09	46.3 (23.8)	38.0 (12.6)	.11	30.8 (41.3)	30.3 (23.8)	≥.99	46.9 (17.1)	48.8 (15.4)	.86	35.4 (25.4)	39.8 (27.8)	.35
Maximum grip strength, kg	27.1 (7.9)	28.3 (8.0)	.63	36.5 (9.9)	35.4 (9.1)	.20	31.6 (8.2)	32.6 (7.4)	.73	31.7 (11.3)	33. 8 (11.5)	.50	45.6 (5.6)	43.2 (7.1)	.56	38.0 (12.6)	37.1 (11.4)	.36	22.5 (6.1)	22.9 (1.6)	≥.99	27.5 (3.3)	27.5 (1.9)	≥.99	27.8 (2.3)	29.9 (4.6)	.70

a discontinuation group (hypertension, 2 persons; dyslipidemia, 1 person)

b diet-only group (diabetes, 1 person; hypertension, 4 persons; dyslipidemia, 1 person).

c diet-plus-exercise group (diabetes, 1 person; hypertension, 4 persons; dyslipidemia, 3 persons; heart disease, 1 person).

d Before-and-after comparison: Wilcoxon signed-rank test.

e ASM: appendicular skeletal mass.

f SMI: skeletal muscle mass index.

## Discussion

In this study, a substantial increase in the SMI was observed in both the diet-only and diet-plus-exercise groups. Previous studies have demonstrated the effectiveness of personalized nutrition plans [5] and interventions that combine exercise and nutrition [6,7]. These studies primarily used nutritional supplements for their experiments. In contrast, this study confirmed the effectiveness of providing regular dietary guidance. These results suggest that interventions based on appropriately customized indirect applications can increase SMI. However, no significant increase in total muscle mass was observed in men in the diet plus exercise group, and no improvement in physical function was observed in either sex. Possible factors include sex differences in trunk and limb muscle strength [8], the small number of participants, and the fact that the exercise program of the application focused on the limbs. Furthermore, low- to moderate-intensity endurance training for older adults typically shows physiological responses after 4 months [9] and is recommended to be performed two to three times per week [10], suggesting that the trial period of the application was too short. Future challenges include expanding the sample size, investigating the intervention period, frequency, and its effects on muscle quality. Although this was a small-scale study without a control group, the results suggest that the program is useful for preventing or improving sarcopenia in older adults.

## Abbreviations

IoT: Internet of Things
SMI: Skeletal Muscle Mass Index
